# Differential prognostic burden of cardiovascular disease and lower-limb amputation on the risk of all-cause death in people with long-standing type 1 diabetes

**DOI:** 10.1186/s12933-022-01487-8

**Published:** 2022-05-09

**Authors:** Marion Camoin, Gilberto Velho, Pierre-Jean Saulnier, Louis Potier, Yawa Abouleka, Charlyne Carpentier, Severine Dubois, Alice Larroumet, Vincent Rigalleau, Elise Gand, Olivier Bourron, Lyse Bordier, André Scheen, Samy Hadjadj, Ronan Roussel, Michel Marre, Kamel Mohammedi

**Affiliations:** 1 Department of Endocrinology, Diabetes and Nutrition, CEDEX, Bordeaux University Hospital, Hôpital Haut-Lévêque, Avenue de Magellan, 33604 Pessac, France; 2 Service d’Endocrinologie Diabétologie Nutrition, Hôpital Bichat, Fédération de Diabétologie de Paris, AP-HP, Université de Paris, Paris, France; 3 INEM, INSERM, Université de Paris, Paris, France; 4 UFR de Médecine et Pharmacie, Université de Poitiers, Poitiers, France; 5 Centre d’Investigation Clinique, CHU de Poitiers, Poitiers, France; 6 Inserm, CIC 1402, Poitiers, France; 7 Service d’Endocrinologie Diabétologie Nutrition, CHU d’Angers, Angers, France; 8 Faculty of Medicine, University of Bordeaux, Bordeaux, France; 9 INSERM U1219, Bordeaux Population Health Research Center, Bordeaux, France; 10 Service de Diabétologie et Métabolisme, APHP, Groupe Hospitalier La Pitié-Salpêtrière, Sorbonne Université, Paris, France; 11 INSERM, UMRS 1138, Centre de Recherche des Cordeliers, Sorbonne Université, Paris, France; 12 Service d’Endocrinologie, Hôpital Bégin, Saint Mandé, France; 13 CHU Liège, Liège Université, Liège, Belgique; 14 Institut du Thorax, INSERM, CNRS, UNIV Nantes, CHU Nantes, Nantes, France; 15 Clinique Ambroise Paré, Neuilly-sur-Seine, France; 16 Biology of Cardiovascular Diseases, INSERM Unit 1034, Pessac, France

**Keywords:** Lower-limb amputation, Cardiovascular disease, Mortality, Myocardial infarction, Stroke, Type 1 diabetes mellitus

## Abstract

**Background:**

Cardiovascular disease (CVD) and nontraumatic lower-limb amputation (LLA) each results in reduced life expectancy in patients with type 1 diabetes, but the differential burden between these conditions is unknown. We compared the effects of CVD and LLA on the risk of mortality in people with type 1 diabetes.

**Methods:**

We used pooled data from the SURGENE, GENEDIAB, and GENESIS prospective cohorts. Data were divided into: 1/absence of CVD (myocardial infarction and/or stroke) nor LLA, 2/history of CVD alone without LLA, 3/LLA alone without CVD or 4/both conditions at baseline. Participants with baseline history of peripheral artery disease were excluded from groups 1 and 2. The study endpoint was any death occurring during follow-up, regardless of the causes.

**Results:**

Among 1169 participants (male 55%, age 40 ± 13 years, diabetes duration 23 ± 11 years), CVD, LLA or both were present at baseline in 49 (4.2%), 62 (5.3%) and 20 (1.7%) subjects, respectively. All-cause death occurred in 304 (26%) participants during 17-year follow-up, corresponding to 18,426 person-years and an incidence rate of 16 (95%CI, 15–18) per 1000 person-years. The risk of death increased in individuals with baseline history of CVD (adjusted HR 2.00 [95% CI 1.34–3.01], p = 0.0008) or LLA (2.26 [1.56–3.28], p < 0.0001), *versus* no condition, with an additive effect in people with both conditions (5.32 [3.14–9.00], p < 0.0001). No incremental risk of death was observed in people with CVD *versus* LLA (0.87 [0.54–1.41]). Compared with no condition, CVD and LLA were similarly associated with reduced life expectancy during follow-up: 2.79 (95% CI 1.26–4.32) and 3.38 (1.87–4.88) years, respectively. Combined conditions expose to 7.04 (4.76–9.31) less years of life expectancy (all p < 0.0001).

**Conclusions:**

CVD and LLA conferred a similar burden regarding mortality in type 1 diabetes population. Our findings encourage a careful consideration of people with type 1 diabetes and LLA as usually recommended for those with CVD, in terms of management of risk factors, treatments and prevention.

**Supplementary Information:**

The online version contains supplementary material available at 10.1186/s12933-022-01487-8.

## Background

Type 1 diabetes is still associated with increased risk of premature mortality as compared to matched individuals from the general population, despite success in reducing risk of diabetes complications over the last decades [1–3]. Cardiovascular disease (CVD) is at least 3-times more prevalent in people with type 1 diabetes, compared with individuals without diabetes [4]. It is well established that CVD accounts for most of the premature deaths occurring in patients with type 1 diabetes [5, 6].

Nontraumatic lower-limb amputations (LLA) is a common complication, with a much higher prevalence in people with type 1 diabetes than in persons without diabetes [7]. It is a devastating complication, associated with major disability, worsening quality of life and considerable impact on health care systems [8–10]. LLA is also associated with a significant reduction in life expectancy, which is usually attributable to coexisting CVD [11, 12]. Beyond increasing the risk of cardiovascular events, LLA is a sentinel outcome, because risk is affected by many conditions, especially peripheral artery disease (PAD), peripheral diabetic neuropathy, diabetic foot ulceration, and infectious disease [8, 13, 14]. The relationship between CVD, LLA and death has not been widely evaluated in people with type 1 diabetes, and fragmentary data have been extrapolated in a large part from studies in population with type 2 diabetes [15–18]. Notably, the difference in the lethal burden induced by CVD or LLA in terms of mortality is still unknown in people with type 1 diabetes. In the current study, we explored whether there is a prognostic difference between a history of CVD and non-traumatic LLA in respect of risk of all-cause death in people with long-standing type 1 diabetes.

## Methods

### Study participants

We used data from three French and Belgian prospective cohorts of people with type 1 diabetes [19–21]. The three studies complied with the Declaration of Helsinki, and the study protocol of each cohort was approved by the Ethics Committee of Angers University Hospital (Angers, France). All participants from the 3 cohorts gave written informed consent. Characteristics of participants at baseline in each single cohort were previously published [22–25], and are shown in Additional Table 1. Briefly, the Survival Genetic Nephropathy (SURGENE) study was a single center, prospective cohort of all volunteers with type 1 diabetes attending the diabetes clinic at the university hospital of Angers, France [19]. Participants were selected from 1989 to 1996 based on a diagnosis of type 1 diabetes before the age of 40 years, and a duration of diabetes longer than 3 years. The *Génétique de la Néphropathie Diabétique* (GENEDIAB) study was a multi-center cohort conducted in 17 diabetes clinics in France and Belgium (see list of centers in Additional File 1). Participants were recruited from May 1994 to April 1995 based on the diagnosis of type 1 diabetes before the age of 35 years, duration of diabetes of at least 5 years, with a past or present history of pre-proliferative or proliferative diabetic retinopathy requiring laser photocoagulation therapy [20]. The Genesis France-Belgique study was a family-based cohort including probands with type 1 diabetes for at least 5 years [21]. Participants were recruited from November 1998 to December 2000 on the basis of a diagnosis of type 1 diabetes before the age of 35 years, with initial ketosis and requirement for permanent insulin treatment within 1 year of diagnosis, and past or present diagnosis of diabetic retinopathy. Participants were followed from enrolment in each corresponding cohort until death or the latest clinical visit up to May 31, 2019. Data from the 3 cohorts were pooled together for the current analysis. Among 1347 participants enrolled in the 3 cohorts, we excluded 35 participants without LLA data at baseline and 98 subjects without a known vital status or follow-up data within the study period (Additional file 4: Fig. S1). We also excluded 45 participants according to the definition of the exposure (see below).

**Table 1:** Characteristics of participants by history of CVD and LLA at baseline

	All participants	History of CVD and/or LLA at baseline					P
		Absent		CVD only	LLA only	Both CVD and LLA	
N	1169	1038		49 (4.2)	62 (5.3)	20 (1.7)	
Cohort membership, n (%)							< 0.0001
SURGENE	337 (29)	330 (32)		5 (10)	2 (3)	0 (0)	
GENEDIAB	376 (32)	284 (27)		22 (45)	54 (87)	16 (80)	
GENESIS	456 (39)	424 (41)		22 (45)	6 (10)	4 (20)	
Male sex, n (%)	643 (55)	552 (53)		32 (65)	41 (66)	18 (90)	0.0009
Age (years)	40 ± 13	39 ± 12		52 ± 10^a^	50 ± 12^a^	51 ± 10^a^	< 0.0001
Age of diabetes onset (years)	15 (10, 23)	14 (10, 23)		19 (11, 29)	18 (12, 25)	16 (11, 24)	0.04
Duration of diabetes (years)	23 ± 11	22 ± 11		31 ± 9^a^	32 ± 9^a^	34 ± 7^a^	< 0.0001
Body mass index (kg/m^2^)	24 ± 3	24 ± 3		25 ± 4	24 ± 4	24 ± 3	0.20
Tobacco smoking, n (%)							< 0.0001
Former	119 (10)	91 (9)		9 (18)	15 (24)	4 (20)	
Current	241 (21)	210 (20)		8 (16)	14 (23)	9 (45)	
Systolic blood pressure (mmHg)	132 ± 19	131 ± 18		140 ± 18^a^	146 ± 22^a^	143 ± 13^a^	< 0.0001
Diastolic blood pressure (mmHg)	76 ± 11	76 ± 11		78 ± 9	81 ± 10^a^	81 ± 8^a^	0.0001
HbA1c (%)	8.8 ± 1.8	8.8 ± 1.8		8.7 ± 1.1	9.2 ± 2.7	8.5 ± 1.5	0.33
HbA1c (mmol/mol)	72 ± 20	72 ± 20		71 ± 12	77 ± 29	70 ± 16	
Total cholesterol (mmol/l)^1^	5.6 ± 1.4	5.6 ± 1.4		6.2 ± 1.4^a^	5.7 ± 1.4	6.3 ± 1.3^a^	0.04
eGFR (mL/min/1.73m^2^)	86 ± 30	89 ± 28		66 ± 31^a^	64 ± 32^a^	61 ± 34^a^	< 0.0001
Urinary albumin concentration (mg/l)	14 (6, 94)	13 (5, 77)		17 (6, 501)	91 (16, 457)	381 (36, 805)	< 0.0001
Diabetic kidney disease, n (%)	484 (41)	398 (38)		26 (53)	42 (68)	18 (90)	< 0.0001
Diabetic retinopathy stages, n (%)							< 0.0001
Non-proliferative	276 (24)	265 (26)		7 (14)	1 (2)	3 (15)	
Pre-proliferative	182 (16)	160 (15)		9 (18)	10 (16)	3 (15)	
Proliferative	503 (43)	405 (39)		33 (67)	51 (82)	14 (70)	
Peripheral diabetic neuropathy, n (%)	412 (35)	314 (30)		29 (59)	51 (82)	18 (90)	< 0.0001
Peripheral artery disease, n (%)	71 (8)	0		0	52 (88)	19 (100)	< 0.0001
Antihypertensive drugs, n (%)	469 (40)	371 (36)		39 (80)	42 (68)	17 (85)	< 0.0001
Lipid-lowering drugs, n (%)	69 (6)	52 (5)		9 (18)	4 (6)	4 (20)	0.0002

Quantitative variables are presented as mean ± SD or as median (25th–75th percentiles) for those with skewed distribution (age of diabetes onset and urinary albumin concentration)

Comparisons were performed using χ2, ANOVA or Kruskal–Wallis tests. Post-hoc test was performed following ANOVA to determine significantly different values compared to subjects without condition (**a**). P < 0.05 was considered as significant

*CVD* cardiovascular disease (myocardial infarction and/or stroke), *LLA* lower-limb amputation

^1^Data available for 664 participants

### Definition of the exposure

Participants were categorized into 4 groups according to the history of CVD (myocardial infarction and/or stroke) and non-traumatic LLA at baseline: (1) people without CVD nor LLA, (2) those with CVD who had not LLA, (3) those with LLA without CVD, and (4) those with both CVD and LLA. To reduce the potential confounding effect related to PAD, participants with a baseline history of PAD in group 1 (n = 37) and group 2 (n = 8) were excluded from the current analysis.

The presence of LLA, myocardial infarction and stroke was reported at baseline in the case report form by using a dedicated questionnaire. An independent adjudication committee validated these conditions based on medical and surgical reports. Myocardial infarction was diagnosed based on the presence of clinical signs, abnormalities in electrocardiogram, and elevated enzymatic biomarkers. Stroke was diagnosed based on abnormal neurological examination and/or cerebral CT Scan evidence. The history of LLA at baseline was defined as either a minor (below the ankle amputation consisting of at least 1-ray metatarsal resection) or major (above the ankle amputation consisting of transtibial or transfemoral) amputation resulting from nontraumatic causes.

### Definition of other clinical conditions at baseline

Peripheral diabetic neuropathy was diagnosed based on the absence of Achilles reflex and the loss of the 10-g monofilament sensation and/or the loss of vibration perception. Lower-limb PAD was defined as the absence of foot pulses and/or intermittent claudication. Diabetic retinopathy was staged as absent, non-proliferative, and proliferative. The Chronic Kidney Disease Epidemiology Collaboration (CKD-EPI) creatinine equation was applied to estimate the glomerular filtration rate (eGFR). History of diabetic kidney disease (DKD) was defined as eGFR < 60 mL/min/1.73 m^2^ and/or urinary albumin concentration (UAC) ≥ 30 mg/l.

### Study endpoint

The endpoint was any death occurring during follow-up, regardless of the causes. The vital status was obtained from hospital case records or by contacting the family physician of the participants. It was cross-checked by contacting the civil registry of the birth place of participants. Death cases were centrally reviewed by an independent adjudication committee by using hospitalization records or all other relevant supporting documents.

Data regarding causes of deaths were available only in a subset of participants. Cardiovascular mortality was defined as deaths resulting from arrythmia, acute myocardial infarction, heart failure, stroke, cardiovascular haemorrhage, PAD, end-stage kidney disease, dead in bed syndrome, sudden deaths or undetermined causes. Non cardiovascular mortality was defined as deaths resulting from cancer, infectious, pulmonary, gastrointestinal, hepatobiliary or pancreatic disease, metabolic complications, suicide, drug reaction or overdose, and trauma or unintentional injuries.

### Statistical methods

Quantitative variables were expressed as mean ± SD or median (25th, 75th percentiles) for variables with skewed distribution. Categorical variables were presented as the number of participants with the corresponding percentage. Chi-squared, ANOVA, Wilcoxon or Kruskal–Wallis tests were used to compare key characteristics of participants at baseline.

Kaplan–Meier curves were used to plot survival rates during follow-up according to the exposure (absence of CVD and LLA, CVD only, LLA only or both CVD and LLA). Incidence curves were compared using the log-rank test. We fitted Cox proportional hazards survival regression models to estimate hazard ratios (HR), with associated 95% CI, for all-cause death in participants with CVD, LLA or both, compared to those without these conditions at baseline. Analyses were adjusted for age, sex and cohort membership (model 1), plus other relevant confounding variables: history of tobacco smoking, duration of diabetes, HbA1c, BMI, systolic blood pressure, UAC, eGFR, and use of antihypertensive and lipid-lowering drugs at baseline (model 2). The Schoenfeld residuals method was used to check the proportional hazards assumption for the association between exposure and all-cause death.

We have also estimated the loss in life-time expectancy within study period in participants with baseline history of CVD, LLA or both conditions, compared to those without these conditions, using the pseudo-observation approach on survival data [26, 27].

We performed a series of sensitivity analyses. First, we evaluated the association between the exposure and the endpoint after adjusting for total cholesterol and history of diabetic peripheral neuropathy and retinopathy stages at baseline (on top of model 2) in a subset of participants from whom these data were available. Second, we assessed the risk of all-cause death according to baseline history of CVD and/or LLA using an alternative definition: without exclusion of participants with a baseline history of PAD in groups 1 and 2. Third, we tested the association of interest considering the baseline history of DKD in the exposure definition. Hence, participants were categorized into 5 groups: (1) people without DKD, CVD or LLA; (2) those with DKD, without CVD or LLA; (3) those with CVD without DKD or LLA; (4) those with LLA without CKD or CVD; and (5) those with 2 conditions or more. Finally, we assessed the association between baseline history of CVD or LLA and the risk of cardiovascular death in a subset of participants from whom causes of death were documented (see Additional file 2, 3).

A p value less than 0.05 was considered as significant. Statistical analyses were performed using SAS software, version 9.4 (SAS Institute, www.sas.com) and Stata software, version 13 (StataCorp, www.stata.com).

## Results

### Characteristics of participants at baseline

Among 1169 participants, 643 (55%) subjects were men, and 31% former or current tobacco smokers. The mean ± SD age, duration of diabetes, HbA1c, systolic and diastolic blood pressure were 40 ± 13 years, 23 ± 11 years, 8.8 ± 1.8%, 132 ± 19 and 76 ± 11 mmHg, respectively (Table 1). A history of CVD and/or LLA was present at baseline in 131 (11.2%) participants: CVD only 49 (4.2%), LLA only 62 (5.3%) and both CVD and LLA 20 (1.7%). Characteristics of participants according to these conditions are shown in Table 1. Participants with a baseline history of CVD or/and LLA, compared to those without these conditions, were older, more frequently men and smokers (Table 1). They had a longer duration of diabetes, higher systolic and diastolic blood pressure, total cholesterol and UAC, and a lower eGFR. They were also more likely to have a history of DKD, proliferative retinopathy, or peripheral neuropathy, and to use antihypertensive or lipid-lowering drugs (Table 1). Baseline history of PAD was present in 88% participants with LLA alone and 100% of those with both CVD and LLA. By study design, PAD was absent in participants without CVD nor LLA and in those with only CVD.

### Incidence of all-cause death during follow-up

Three hundred and four (26%) patients died during a median (25th, 75th percentiles) duration of follow-up of 17 (10–22) years, corresponding to 18,426 person-years and an incidence rate of 16 (95% CI, 15–18) per 1000 person-years. Characteristics of participants at baseline according to the incidence of all-cause death are shown in Table 2. Patients who died, compared with those who were still alive within study period, were older at baseline, and more frequently men (Table 2). They had a longer duration of diabetes, higher systolic and diastolic blood pressure, total cholesterol, and UAC, and a lower eGFR. The baseline history of tobacco smoking, DKD, proliferative retinopathy, peripheral neuropathy or PAD, and the use of antihypertensive or lipid-lowering drugs were more frequent in patients who died, compared with those who were still alive during follow-up (Table 2).

**Table 2:** Characteristics of participants at baseline by the incidence of all-cause death during follow-up

	All-cause death		P
	No	Yes	
N	865	304	
Cohort membership, n (%)			< 0.0001
SURGENE	275 (32)	62 (20)	
GENEDIAB	212 (25)	164 (54)	
GENESIS	378 (44)	78 (26)	
Male sex, n (%)	452 (52)	191 (63)	0.001
Age (years)	37 ± 11	49 ± 13	< 0.0001
Age of diabetes onset (years)	14 (10, 22)	17 (12, 27)	< 0.0001
Duration of diabetes (years)	21 ± 11	30 ± 10	< 0.0001
Body mass index (kg/m2)	24 ± 3	24 ± 4	0.36
Tobacco smoking, n (%)			< 0.0001
Former smokers	69 (8)	50 (16)	
Current smokers	165 (19)	76 (25)	
Systolic blood pressure (mmHg)	129 ± 18	141 ± 19	< 0.0001
Diastolic blood pressure (mmHg)	75 ± 11	79 ± 11	< 0.0001
HbA1c (%)	8.8 ± 1.8	8.8 ± 1.9	0.52
HbA1c (mmol/mol)	72 ± 20	73 ± 21	
Total cholesterol (mmol/l)^a^	5.5 ± 1.4	5.9 ± 1.5	0.0003
eGFR (mL/min/1.73m^2^)	92 ± 27	70 ± 31	< 0.0001
Urinary albumin concentration (mg/l)	11 (5, 51)	45 (8, 552)	< 0.0001
Diabetic kidney disease, n (%)	299 (35)	185 (61)	< 0.0001
Diabetic retinopathy stages, n (%)			< 0.0001
Non-proliferative	242 (28)	34 (11)	
Pre-proliferative	123 (14)	59 (19)	
Proliferative	307 (35)	196 (64)	
Peripheral diabetic neuropathy, n (%)	246 (28)	166 (55)	< 0.0001
Peripheral artery disease, n (%)	16 (3)	55 (23)	< 0.0001
Antihypertensive drugs, n (%)	283 (33)	186 (62)	< 0.0001
Lipid-lowering drugs, n (%)	37 (4)	32 (10)	< 0.0001

Quantitative variables are presented as mean ± SD or as median (25th–75th percentiles) for those with skewed distribution (age of diabetes onset and urinary albumin concentration)

Comparisons were performed using χ2, ANOVA or Wilcoxon tests

P < 0.05 was considered as significant

^a^Data available for 664 participants

### Incidence of all-cause death by baseline history of CVD and/or LLA

**Fig. 1:**
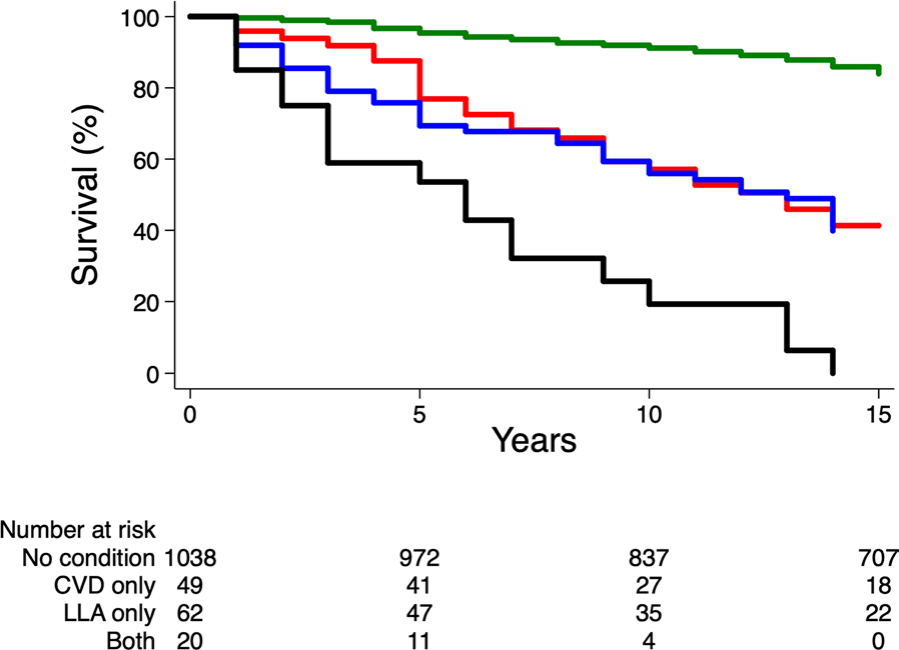
Kaplan Meier curves estimating the survival according to baseline history of cardiovascular disease (CVD) and lower-limb amputation (LLA): absent (green line), CVD only (red line), LLA only (blue line), and both CVD and LLA (black line). P < 0.0001

The cumulative incidence and the incidence rates of all-cause death were significantly higher in participants with a baseline history of CVD, LLA or both, compared to those without these conditions (Fig. 1 and Table 3). The relative risk of all-cause death was higher in participants with CVD (HR 2.47 [95% CI 1.67–3.66]), LLA (2.38 [1.68–3.38]) or both conditions (8.50 [5.09–14.19]), compared to those without these conditions at baseline, after adjusting for age, sex and cohort membership (p < 0.0001 for all analyses). Similar results were observed after adjusting for further confounding variables (model 2, Table 3). No prognostic difference was observed between baseline history of CVD and LLA in regards of risk of all-cause death (CVD versus LLA: HR 0.87 [0.54–1.41], p = 0.57) after adjusting for model 2. The presence of both conditions at baseline, versus CVD alone (HR 2.72, [1.48–4.99], p = 0.001) or versus LLA alone (HR 2.36, [1.34–4.18], p = 0.003), was associated with increased risk of all-cause death.

**Table 3:** Risk of all-cause death during follow-up by history of CVD or/and LLA at baseline

	All-cause death			Model 1		Model 2	
	No, n	Yes, n (%)	Incidence rate (95% CI)	Hazard ratios (95% CI)	P	Hazard ratios (95% CI)	P
Absent	827	211 (20)	12 (11–14)	1	–	1	–
Cardiovascular disease only	19	30 (61)	53 (37–76)	2.47 (1.67–3.66)	< 0.0001	2.00 (1.34–3.01)	0.0008
Lower-limb amputation only	17	45 (73)	61 (45–81)	2.38 (1.68–3.38)	< 0.0001	2.26 (1.56–3.28)	< 0.0001
Both conditions	2	18 (90)	156 (99–248)	8.50 (5.09–14.19)	< 0.0001	5.32 (3.14–9.00)	< 0.0001

Incidence rate expressed per 1000 person-years. Hazard ratios (with associated 95% CIs) computed by Cox proportional hazards survival regression analyses, adjusted for cohort membership, sex and age (model 1), plus history of tobacco smoking, duration of diabetes, BMI, HbA1c, systolic blood pressure, urinary albumin concentration, estimated glomerular filtration rate, and use of antihypertensive and lipid-lowering drugs at baseline (model 2). p < 0.05 was considered as significant

### Sensitivity analyses

#### All-cause death by baseline history of CVD and/or LLA using an extended adjusting model

Associations between baseline history of CVD and/or LLA and all-cause death had similar magnitudes after adjustment for total cholesterol and history of diabetic peripheral neuropathy and retinopathy stages at baseline (further to adjusting for model 2) in 633 participants for whom these data were available: CVD alone (1.75 [1.03–2.96], p = 0.04), LLA alone (1.98 [1.31–3.00], p = 0.001), or both CVD and LLA (4.75 [2.57–8.78], p < 0.0001).

#### All-cause death by baseline history of CVD and/or LLA using an alternative definition (without exclusion of participants with PAD in groups 1 and 2)

A history of CVD only, LLA only and both conditions were present at baseline in 57 (4.7%), 62 (5.1%) and 20 (1.7%) participants, respectively. The risk of all-cause death was significantly higher in participants with a baseline history of CVD only (HR 2.02 [95% CI 1.39–2.94], p = 0.0002), LLA only (2.23 [1.54–3.23], p < 0.0001), or both conditions (5.29 [3.14–8.90], p < 0.0001), compared to those without these conditions.

#### All-cause death by baseline history of DKD, CVD and/or LLA

DKD only, CVD only and LLA only were present at baseline in 423 (35%), 25 (2.1%) and 20 (1.7%) participants, respectively. The risk of all-cause death was similarly increased in participants with a baseline history of DKD only (HR 2.30 [95% CI, 1.75–3.01], p < 0.0001), CVD only (2.50 [1.38–4.52], p = 0.003) and LLA only (2.11 [1.13–3.92], p = 0.02), compared to those without these 3 conditions. The presence of 2 of these conditions or more was observed at baseline in 92 (7.6%) participants, and it was associated with excess risk of death (5.88 [4.21–8.20], p < 0.0001).

#### Incidence of cardiovascular death by baseline history of CVD and/or LLA

Causes of death were documented in 97 patients: 13 participants were excluded according to study flow chart (Additional file 4: Fig. S1), and then 84 cases were investigated in the current analysis. Deaths from cardiovascular and non-cardiovascular causes were reported in 50 (4.3%) and 34 (2.9%) patients, respectively. Cardiovascular causes were more prevalent than non-cardiovascular causes in people with baseline history of CVD and/or LLA (p < 0.0001, Additional Table 2). The baseline history of CVD (HR 2.98 [95% CI, 1.13–7.91], p = 0.03) or LLA (3.81 [1.28–11.32], p = 0.02) was associated with increased risk of cardiovascular mortality.

### Estimated reduction in life-time expectancy

Participants with a history of CVD or LLA, compared to those without these conditions, would have a mean (95% CI) reduction in life-time expectancy during the 17 years of follow-up of 3.32 (1.72–4.92) or 3.96 (2.47–5.45) years, respectively (all p < 0.0001, adjusted for model 1). The presence of both CVD and LLA at baseline was associated with 8.11 (5.95–10.26) less years of life expectancy, compared with the absence of these conditions at baseline (p < 0.0001, adjusted for model 1). The estimation of loss in life-time expectancy during study period was comparable after adjusting for model 2: CVD 2.79 (1.26–4.32), LLA 3.38 (1.87–4.88), and both CVD and LLA 7.04 (4.76–9.31) years (all p < 0.0001).

## Discussion

In the present investigation, we compared the effects of a baseline history of CVD (myocardial infarction and/or stroke), nontraumatic LLA or both on the incidence of all-cause death in people with long-standing type 1 diabetes. As expected, CVD or LLA at baseline was each associated with increased risk of all-cause death during follow-up. The original and the key finding of our study was the observation that a baseline LLA conferred a similar prognostic burden than baseline CVD in terms of all-cause death in patients with long-standing type 1 diabetes. The cumulative incidence of all-cause death was 61% and 73% over a median duration of follow-up of 17 years in participants with baseline history of LLA or CVD, respectively. Each single condition was associated with two-fold increased risk of all-cause death. The burden of LLA and CVD seemed independent, with an additive effect as the presence of both conditions increased the cumulative incidence (90%), and the adjusted relative risk of all-cause death, 5 times higher compared with the absence of these conditions at baseline. Furthermore, the presence of both conditions at baseline increased the risk of all-cause death, more than 2 times higher compared with the presence of each single condition. The excess risk of all-cause death observed in people with CVD and LLA was mainly driven by cardiovascular causes.

### Incidence of all-cause death in people with type 1 diabetes

The incidence of all-cause death observed in our study is comparable with the recent report in people with long-standing (50-year cohort) type 1 diabetes from the Finnish Diabetic Nephropathy Study (FinnDiane) cohort [28]. However, the rate of mortality in our study is higher than other reports in type 1 diabetes cohorts from USA, Europe and Australia [1, 2, 29]. As in FinnDiane 50-year cohort, the participants in our cohorts were older, and they were diagnosed with type 1 diabetes between 1938 and 1995. Furthermore, GENESIS and GENEDIAB cohorts recruited participants with a history of non-proliferative or proliferative retinopathy. Finally, 41% of participants in our cohorts had a baseline history of DKD, which has been recognized as a major predictor of death in patients with type 1 diabetes [30].

It is worthy to note that the cumulative incidence of all-cause death was high (20%) even in people with type 1 diabetes without a baseline history of CVD or LLA and a rather young age of 40 years at baseline (yet with a long duration of type 1 diabetes). This observation is in line with a recent report from a large Swedish diabetes registry highlighting the early onset of type 1 diabetes and the longer duration of diabetes as important determinants of death and cardiovascular events [4]. The participants in our study were diagnosed with type 1 diabetes at 15 (25th, 75th percentiles, 10, 23) years old, with a duration of diabetes at baseline of 23 ± 11 years. A longer duration of diabetes reflects a high glycaemic load and its related damages.

### Effect of cardiovascular disease and lower-limb amputation in life-time expectancy

Our findings show also that CVD and LLA had a similar impact in terms of loss in life-time expectancy. Among people with type 1 diabetes, those with CVD or LLA may lose about 3 years life-time during the 17-year follow-up compared with other participants without these conditions, after adjustment for potential confounders. The presence of both conditions exposes to a 7-year loss in life expectancy within study period. It has been shown that people with type 1 diabetes had a drastic reduction in life expectancy, compared with individuals without diabetes [6, 31]. One study estimated that people with type 1 diabetes had an estimated life expectancy at birth 12 years less than that of the general population, using an Australian national data scheme between 1997 and 2010 [31]. Comparable findings were reported in a Scottish cohort of people with type 1 diabetes based on data from 2008 through 2010 [6]. In our present study, we were able to extend those findings in order to estimate and compare the loss in life-time expectancy among people with type 1 diabetes with versus without CVD and/or LLA, followed up to May 31, 2019.

### Potential mechanisms linking lower-limb amputation to cardiovascular disease

The similar poor prognosis observed in participants with baseline CVD and LLA may be explained by common traditional risk factors and conditions [32]. In our study, participants with CVD and LLA shared key risk factors and major diabetes complications. Microvascular disease may also be a trigger of poor prognosis in people with CVD and LLA, independently of traditional risk factors [33–35]. In our study, eGFR decreased, albuminuria increased, neuropathy became more frequent and retinopathy more severe, proportionally to the burden of baseline history of CVD and LLA (either or both together). Of note, we observed that DKD, CVD and LLA (each considered individually) confer a similar risk of all-cause death, twofold higher compared to the absence of these 3 conditions. This finding suggests that DKD may not explain (at least not fully) the poor prognosis observed in people with CVD or LLA. Furthermore, LLA and CVD may also share some pathological mechanisms including endothelial dysfunction, pro-thrombotic states, oxidative stress and systemic inflammation [24, 36–40]. Autoimmune mechanisms have also been suggested as a potential determinant of CVD in people with type 1 diabetes [41].

### Strengths and limitations

The main strength of our study is the assessment of single, joint and differential prognostic effects of baseline history of CVD and LLA on the risk of all-cause death in three multicenter binational cohorts of patients with long-standing type 1 diabetes followed for a median duration of 17 years. We also investigated a comprehensive set of demographic, clinical, and biological features at baseline, as well as robust and adjudicated conditions at baseline and endpoint during follow-up. Our study has some limitations to acknowledge. The diagnosis of PAD in our study was based on interview and clinical examination, without systematic screening (using specific investigation such us ankle-brachial index) for asymptomatic PAD. Our study does not allow to examine the prognostic burden of PAD independently of LLA since PAD was present at baseline in 88% and 100% in group 3 (participants with LLA alone) and group 4 (both CVD and LLA), respectively. To avoid misinterpretation, we excluded PAD participants from group 1 (without CVD and LLA) and group 2 (with CVD alone). Nevertheless, keeping PAD in these groups did not change our findings. Additionally, we did not have a comprehensive data regarding causes of deaths, which did not allow us to investigate carefully the cause-specific mortality. Finally, our results may not be applied to people with other ethnic backgrounds or low-income and middle-income regions as we investigated only Caucasians from France and Belgium.

## Conclusion

In summary, a baseline history of CVD and LLA had a similar and heavy burden regarding all-cause death in people with long-standing type 1 diabetes. Each single condition exposes to two-fold increased risk of all-cause death, with an additive effect. The presence of both CVD and LLA at baseline exposed to exceedingly excess risk of death, five times higher compared to the absence of these conditions. Our findings encourage a careful consideration of people with type 1 diabetes and LLA as usually recommended for CVD, in terms of management of risk factors, treatments and prevention.

## Supplementary Information

**Additional file 1.**List of contributors to SURGENE, GENEDIAB, and GENESIS studies.

**Additional file 2: Table S1.**Characteristics of participants at baseline in each individual cohort.

**Additional file 3: Table S2.** Causes of deaths.

**Additional file 4: Fig. S1.** Study flowchart.

## Data Availability

The datasets analysed during the current study are not publicly available due to consideration of intellectual property, due to many ongoing active collaborations, and to continuing analyses by the study investigators, but may be available from the last author on reasonable request.

## Abbreviations

CKD: Chronic kidney disease
CKD-EPI: Chronic kidney disease epidemiology collaboration
CVD: Cardiovascular disease
DKD: Diabetic kidney disease
eGFR: Estimated glomerular filtration rate
FinnDiane: Finnish Diabetic Nephropathy Study
GENEDIAB: *Génétique de la Néphropathie Diabétique*
LLA: Lower-limb amputation
PAD: Peripheral artery disease
SURGENE: Survival Genetic Nephropathy
UAC: Urinary albumin concentration
