# Supplementary material for: Differential prognostic burden of cardiovascular disease and lower-limb amputation on the risk of all-cause death in people with long-standing type 1 diabetes
Source: Cardiovasc Diabetol. 2022 May 9;21:71. doi: 10.1186/s12933-022-01487-8 (PMC9088124; doi:10.1186/s12933-022-01487-8)
Supplement: Supplementary file 1 — Additional file 1.List of contributors to SURGENE, GENEDIAB, and GENESIS studies. [file 12933_2022_1487_MOESM1_ESM.docx]

**Additional File**

**List of contributors to SURGENE, GENEDIAB, and GENESIS studies**

**Clinical investigators at baseline by alphabetical order of enrollment centers**

All cities are in France, except for Liège, in Belgium:

Albert Fournier, Jean-Daniel Lalau (*Centre Hospitalier Universitaire d’Amiens*); Béatrice Bouhanick, Line Godiveau, Michel Marre, Vincent Rohmer (*Centre Hospitalier Universitaire d’Angers*); Jean-Raymond Attali, Patrick Miossec (*Assistance Publique des Hôpitaux de Paris, Hôpital de Bondy*); Henri Gin, Vincent Rigalleau (*Centre Hospitalier Universitaire de Bordeaux*); Isabelle Cerf, Guillaume Charpentier, Isabelle Petit, Jean-Pierre Riveline (*Centre Hospitalier de Corbeil-Essonne*); Bertrand Godeau, Zoubida Kahal, Dominique Simon (*Assistance Publique des Hôpitaux de Paris, Hôpital de Créteil*); Daniel Cordonnier, Serge Halimi (*Centre Hospitalier Universitaire de Grenoble*); Pierre-Jean Lefebvre, Nicolas Paquot, André Scheen, Laurent Weekers (*Centre Hospitalier Universitaire de Liège*); Pierre Fontaine, Gaëtan Prevot (*Centre Hospitalier Régional Universitaire de Lille*); François Berthezene, Fabrice Bonnet, Maurice Laville, Jean-Pierre Fauvel, Charles Thivolet (*Assistance Publique des Hôpitaux de Lyon*); Bertrand Dussolle, Philippe Vague (*Assistance Publique des Hôpitaux de Marseille*); Patrick Giraud (*Clinique Pont de Chaume, Montauban*); Jacques Bringer, Florence Galtier, Michel Rodier (*Centres Hospitaliers Universitaires de Montpellier et Nîmes*); Pierre Drouin, Laurent Dusselier, Thérèse Crea, Bruno Guerci, Michèle Kessler (*Centre Hospitalier Universitaire de Nancy*); Lucy Chaillous, Bernard Charbonnel (*Centre Hospitalier Universitaire de Nantes*); Hamid Boukersi, Françoise Defrance, Etienne Larger, Michel Marre, Ronan Roussel (*Assistance Publique des Hôpitaux de Paris, Hôpital Bichat-Claude Bernard*); Gérard Slama, Agnès Sola (*Assistance Publique des Hôpitaux de Paris, Hôpital de l’Hôtel Dieu*); André Grimaldi, Agnès Heurtier, Caroline Sert (*Assistance Publique des Hôpitaux de Paris, Hôpital de La Pitié Salpétrière*); Jean-Pierre Grunfeld (*Assistance Publique des Hôpitaux de Paris, Hôpital Necker*); Ahmed Bouallouche, Jean-François Gautier, Pierre-Jean Guillausseau, Hervé Leblanc, Philippe Passa (*Assistance Publique des Hôpitaux de Paris, Hôpitaux Saint-Louis et Lariboisière*); Samy Hadjadj, Richard Maréchaud, Anne Muller, Florence Torremocha (*Centre Hospitalier Universitaire de Poitiers*); Jacques Chanard, Jean-Paul Melin (*Centre Hospitalier Universitaire de Reims*); Hubert Allannic, Jean-Yves Poirier (*Centre Hospitalier Universitaire de Rennes*); Christian Charasse (*Centre Hospitalier de Saint-Brieuc*); Bernard Bauduceau, Lyse Bordier, Hervé Mayaudon (*Hôpital d'Instruction des Armées Bégin, Saint-Mandé*); Marie-Pierre Arpin-Bott (*Centre Hospitalier Universitaire de Strasbour*g); Hélène Hanaire, Pierre Gourdy, Henri Sackmann, Jean-Pierre Tauber (*Centre Hospitalier Universitaire de Toulouse*); Odile Verier-Mine (*Centre Hospitalier de Valenciennes).*
