# Supplementary material for: Differential prognostic burden of cardiovascular disease and lower-limb amputation on the risk of all-cause death in people with long-standing type 1 diabetes
Source: Cardiovasc Diabetol. 2022 May 9;21:71. doi: 10.1186/s12933-022-01487-8 (PMC9088124; doi:10.1186/s12933-022-01487-8)
Supplement: Supplementary file 2 — Additional file 2: Table S1.Characteristics of participants at baseline in each individual cohort. [file 12933_2022_1487_MOESM2_ESM.docx]

**Additional Table 1. Characteristics of participants at baseline in each individual cohort**

|  | **SURGENE** | **GENEDIAB** | **GENESIS** |
| --- | --- | --- | --- |
| N | 337 | 376 | 456 |
| Male sex, n (%) | 191 (57) | 212 (56) | 240 (53) |
| Age (years) | 34 ± 13 | 44 ± 12 | 42 ± 11 |
| Age of diabetes onset (years) | 17 (12, 26) | 14 (9, 21) | 14 (9, 24) |
| Duration of diabetes (years) | 15 ± 11 | 28 ± 10 | 26 ± 9 |
| Body mass index (kg/m^2^) | 23 ± 3 | 24 ± 3 | 24 ± 4 |
| Tobacco smoking*, n (%) | - |  |  |
| Former | - | 68 (18) | 51 (11) |
| Current | - | 105 (28) | 136 (30) |
| Systolic blood pressure (mmHg) | 127 ± 16 | 139 ± 18 | 131 ± 20 |
| Diastolic blood pressure (mmHg) | 73 ± 11 | 80 ± 11 | 76 ± 10 |
| HbA1c (%) | 9.3 ± 2.3 | 8.7 ± 1.8 | 8.5 ± 1.3 |
| HbA1c (mmol/mol) | 79 ± 25 | 71 ± 20 | 69 ± 14 |
| Total cholesterol (mmol/l)^§^ | 5.4 ± 1.4 | 5.6 ± 1.5 | NA |
| eGFR (mL/min/1.73m^2^) | 99 ± 22 | 74 ± 28 | 87 ± 32 |
| Urinary albumin concentration (mg/l) | 7 (4, 14) | 45 (8, 502) | 17 (7, 126) |
| Diabetic kidney disease, n (%) | 57 (17) | 225 (60) | 202 (44) |
| Diabetic retinopathy, n (%) |  |  |  |
| Non-proliferative | 51 (15) | 6 (2) | 219 (48) |
| Pre-proliferative | 43 (13) | 67 (18) | 72 (16) |
| Proliferative | 35 (10) | 303 (81) | 165 (36) |
| Peripheral diabetic neuropathy, n (%)* | 2 (0.6) | 253 (67) | 157 (34) |
| Peripheral artery disease, n (%)* | - | 65 (18) | 6 (1) |
| Antihypertensive drugs, n (%) | 42 (12) | 207 (56) | 220 (48) |
| Lipid-lowering drugs, n (%) | 8 (2) | 33 (9) | 28 (6) |

Quantitative data presented as mean±SD or as median (25^th^–75^th^ percentiles) for those with skewed distribution (age of diabetes onset and urinary albumin concentration).

^§^Data available in a subset of participants (N=664).

*Data not collected comprehensively in SURGENE (smoking, neuropathy) and/or deleted by study design in 45 cases (PAD, see Additional Figure 1).
