# Supplementary material for: Differential prognostic burden of cardiovascular disease and lower-limb amputation on the risk of all-cause death in people with long-standing type 1 diabetes
Source: Cardiovasc Diabetol. 2022 May 9;21:71. doi: 10.1186/s12933-022-01487-8 (PMC9088124; doi:10.1186/s12933-022-01487-8)
Supplement: Supplementary file 3 — Additional file 3: Table S2. Causes of deaths. [file 12933_2022_1487_MOESM3_ESM.docx]

**Additional Table 2. Causes of deaths**

|  | **History of CVD and/or LLA at baseline** | | | |
| --- | --- | --- | --- | --- |
|  | **Absent** | **CVD only** | **LLA only** | **Both CVD and LLA** |
| Alive | 827 (80) | 19 (39) | 17 (27) | 2 (10) |
| Cardiovascular deaths | 37 (4) | 5 (10) | 5 (8) | 3 (15) |
| Non cardiovascular deaths | 31 (3) | 2 (4) | 1 (2) | 0 |
| Deaths with unknown causes | 143 (14) | 23 (47) | 39 (63) | 15 (75) |

Data are expressed as the number of patients (with the corresponding percentage)
