# Supplementary material for: Differential prognostic burden of cardiovascular disease and lower-limb amputation on the risk of all-cause death in people with long-standing type 1 diabetes
Source: Cardiovasc Diabetol. 2022 May 9;21:71. doi: 10.1186/s12933-022-01487-8 (PMC9088124; doi:10.1186/s12933-022-01487-8)
Supplement: Supplementary file 4 — Additional file 4: Fig. S1. Study flowchart. [file 12933_2022_1487_MOESM4_ESM.pdf]

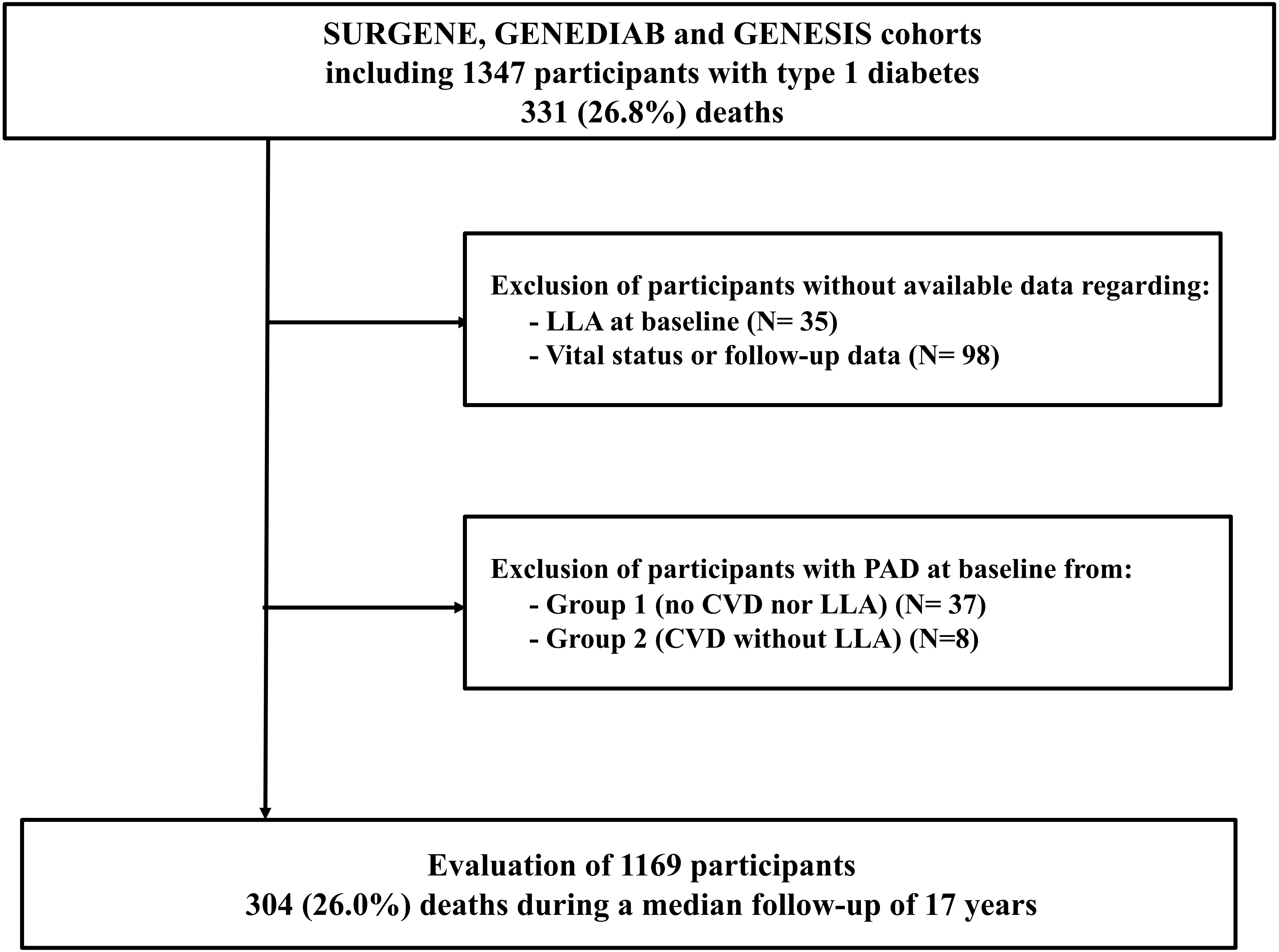
SURGENE, GENEDIAB and GENESIS cohorts
including 1347 participants with type 1 diabetes
331 (26.8%) deaths
Exclusion of participants without available data regarding:
- LLA at baseline (N= 35)
- Vital status or follow-up data (N= 98)
Exclusion of participants with PAD at baseline from:
- Group 1 (no CVD nor LLA) (N= 37)
- Group 2 (CVD without LLA) (N=8)
Evaluation of 1169 participants
304 (26.0%) deaths during a median follow-up of 17 years
